# Way forward and next steps in European Mental Health Policies

**DOI:** 10.1192/j.eurpsy.2025.99

**Published:** 2025-08-26

**Authors:** A. Fiorillo

**Affiliations:** University of Campania “Luigi Vanvitelli”, Naples, Italy

## Abstract

**Abstract:**

The EPA Action Plan 2025-2027 aims to optimize mental health care in Europe, by focusing on several unmet needs still experienced by patients. New ways for treatment delivery and new settings of care, tailored diagnostic approaches, research on brain and on mental health, reduction of multimorbidity and mortality gap, promotion of prevention strategies in mental health and more attention on minorities’ mental health represent some of the milestones on which a roadmap for mental health in Europe may be based.

**Disclosure of Interest:**

None Declared

